# Coenzyme Q10 therapy in patients with post COVID-19 condition – Authors’ response

**DOI:** 10.1016/j.lanepe.2022.100571

**Published:** 2023-01-18

**Authors:** Kristoffer Skaalum Hansen, Steffen Leth

**Affiliations:** a Department of Infectious Diseases, Aarhus University Hospital, Aarhus, Denmark; b Department of Clinical Medicine, Aarhus University, Aarhus, Denmark; c Department of Infectious Diseases & Internal Medicine, Gødstrup Regional Hospital, Herning, Denmark

We appreciate Kow and colleagues’ relevant comment^1^ to the study on the effect of coenzyme Q10 in post COVID-19 condition (PCC).^2^ Among other hypotheses, the cytokine profile of patients with PCC and the role of interleukin – especially interleukin-6 (IL-6) – and its interaction with the mitochondrion is an area of high importance in the search of a pathogenesis of PCC.

The hypothesis presented by Kow et al. is interesting. It should be mentioned, that in the preprint meta-analysis by Yin et al. four studies comparing the levels of serum IL-6 in long COVID and previously SARS-CoV-2 infected without long COVID were included. Long COVID here is defined as 28 days after onset, unlike PCC, which is persisting symptoms after 12 weeks.^3^^,^^4^

A subgroup analysis on a cohort with BMI less than 26 was performed. The number of participants with BMI <26 was 51. In this sub-cohort, mean BMI was now 23.8, mean age 46 years and 39 (76.5%) were female. Group A and B consisted of 25 and 28 participants, respectively, and were still well-balanced. There was no significant difference between the subgroup of participants with BMI <26 and the original cohort with regards to baseline parameters (other than BMI).

There was no significant difference in reduction of PCC symptom score (−0.002 (95% CI -3.64; 3.66), P = 0.99) or increase in EQ-5D health index (−0.006 (95% CI -0.44; 0.032), P = 0.75) when comparing CoQ10 and placebo with a mixed-effect linear regression model in this subgroup. A subsequent analysis on symptom clusters did not show any significant difference between CoQ10 and placebo (Fig. 1). Importantly, subgroup analysis results should be interpreted with caution due to the risk of false positive by multiple comparisons and – especially in this case – limited power after reduction of the number of observations. Thus, larger cohort studies are needed to establish a connection between mitochondrial dysfunction in PCC and the involvement of interleukins.

**Fig. 1:**
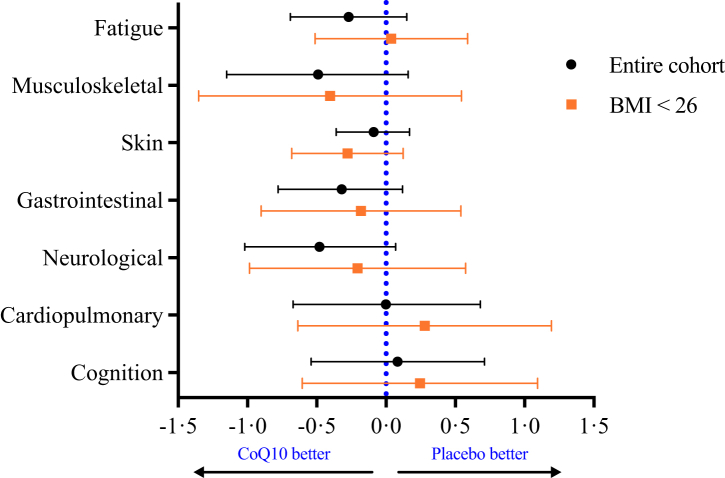
**Results of an additional mixed-effects model analysis for seven symptom groups derived from the PCC-specific questionnaire comparing treatment effect in entire cohort versus a subgroup with BMI less than 26.** The mean regression coefficients (slopes) with a 95% confidence interval are plotted to visualize the difference in the change in symptom scores between CoQ10 and placebo. A negative slope indicates a larger reduction in the symptom score with CoQ10 compared to placebo; the dotted vertical line at 0.0 indicates no difference between CoQ10 and placebo. Orange circles represent the subgroup with BMI <26 (n = 51) while black circles represent the entire cohort (n = 119). None of the estimated difference in the symptom groups reached statistical significance.

## Contributors

Kristoffer Skaalum Hansen performed the subgroup analysis and wrote the correspondence. Steffen Leth critically revised the correspondence.

## Declaration of interests

We declare no competing interests.
